# Patient characteristics associated with being offered take home naloxone in a busy, urban emergency department: a retrospective chart review

**DOI:** 10.1186/s12913-019-4469-3

**Published:** 2019-09-05

**Authors:** Daniel C. O’Brien, Daniel Dabbs, Kathryn Dong, Paul J. Veugelers, Elaine Hyshka

**Affiliations:** 1 University of Alberta, School of Public Health, 3-300 Edmonton Clinic Health Academy, 11405 – 87 Ave, Edmonton, Alberta T6G 1C9 Canada; 2 University of Alberta, Faculty of Medicine and Dentistry, 2J2.00 WC Mackenzie Health Sciences Centre, 8440 112 St. NW, Edmonton, Alberta T6G 2R7 Canada; 3 University of Alberta, Faculty of Medicine and Dentistry, 790 University Terrace Building, 8303 112 St. NW, Edmonton, Alberta T6G 2T4 Canada; 4 University of Alberta, School of Public Health, 33-50 University Terrace, 8303 - 112 Ave, Edmonton, Alberta T6G 2T4 Canada

**Keywords:** Take home naloxone, Emergency department, Opioids, Overdose

## Abstract

**Background:**

Overdose deaths can be prevented by distributing take home naloxone (THN) kits. The emergency department (ED) is an opportune setting for overdose prevention, as people who use opioids frequently present for emergency care, and those who have overdosed are at high risk for future overdose death. We evaluated the implementation of an ED-based THN program by measuring the extent to which THN was offered to patients presenting with opioid overdose. We analyzed whether some patients were less likely to be offered THN than others, to identify areas for program improvement.

**Methods:**

We retrospectively reviewed medical records from all ED visits between April 2016 and May 2017 with a primary diagnosis of opioid overdose at a large, urban tertiary hospital located in Alberta, Canada. A wide array of patient data was collected, including demographics, opioid intoxicants, prescription history, overdose severity, and whether a naloxone kit was offered and accepted. Multivariable analyses were used to identify patient characteristics and situational variables associated with being offered THN.

**Results:**

Among the 342 ED visits for opioid overdose, THN was offered in 49% (*n* = 168) of cases. Patients were more likely to be offered THN if they had been found unconscious (Adjusted Odds Ratio 3.70; 95% Confidence Interval [1.63, 8.37]), or if they had smoked or injected an illegal opioid (AOR 6.05 [2.15,17.0] and AOR 3.78 [1.32,10.9], respectively). In contrast, patients were less likely to be offered THN if they had a current prescription for opioids (AOR 0.41 [0.19, 0.88]), if they were admitted to the hospital (AOR 0.46 [0.22,0.97], or if they unexpectedly left the ED without treatment or before completing treatment (AOR 0.16 [0.22, 0.97).

**Conclusions:**

In this real-world evaluation of an ED-based THN program, we observed that only half of patients with opioid overdose were offered THN. ED staff readily identify patients who use illegal opioids or experience a severe overdose as potentially benefitting from THN, but may miss others at high risk for future overdose. We recommend that hospital EDs provide additional guidance to staff to ensure that all eligible patients at risk of overdose have access to THN.

## Background

Opioid overdose is a leading, yet preventable cause of death in North America and around the world [1, 2]. Naloxone, an opioid antagonist, is commonly used in clinical settings to reverse the potentially lethal respiratory depression that occurs during opioid overdose [3]. “Take Home Naloxone” (THN) programs aim to prevent deaths by distributing naloxone to people likely to witness an opioid overdose, such as people who use drugs and their family and friends [4–6]. Typically, THN programs train people to recognize the signs of an opioid overdose and respond appropriately by providing basic life support and administering naloxone via either intranasal spray or intramuscular injection [2, 5, 6]. The World Health Organization has identified THN distribution as a key health intervention to prevent opioid overdose deaths [2].

Research on THN programs has shown that THN kits are frequently used by people who use drugs to respond to overdoses [7, 8]. Specifically, approximately 25% of people who use drugs who are trained and supplied with naloxone will use it to reverse an overdose within 1 year [7]. At the population-level, reductions in overdose mortality have been observed following the implementation of THN programs, and higher rates of kit distribution lead to greater reductions in mortality [9, 10]. THN distribution can be particularly effective at reducing overdose mortality when targeted at high risk populations, such as recently released prisoners who have lost their opioid tolerance [11].

In recent years, THN programs have been increasingly incorporated into hospital Emergency departments (EDs) in an effort to reach high risk patients [10, 12]. Hospital EDs provide a critical opportunity to reach people at risk for overdose, because people who use opioids frequently present for emergency care, and those who have overdosed are at high risk of future overdose death [13–15]. Indeed, ED visits may be one of the few occasions that an individual comes in contact with the health system before experiencing a fatal overdose. Previous investigations have demonstrated that ED-based THN distribution is feasible [16, 17], and that the majority of clinicians are willing to provide THN in the ED. [18, 19] Further, approximately 70% of at-risk ED patients who are offered THN accept it [20].

For ED-based THN programs to have the greatest impact, THN should be offered to all patients who are at risk of overdose, including those who report using illegal opioids, those taking high doses of prescribed opioids, and those using opioids who present with complications other than opioid overdose (e.g. abscesses, trauma, etc.) [20–22]. However, ensuring all ED patients at risk of overdose are offered naloxone may be challenging [22, 23]. For instance, one evaluation found that THN was offered to only 8% of ED patients with International Classification of Disease codes for opioid overdose, misuse, or dependence [23]. In previous qualitative studies, ED providers who were asked to identify barriers to providing THN reported lacking clarity about which patients should be offered naloxone [23, 24]. This is problematic, because correctly identifying at-risk patients to offer THN is a crucial step in providing THN to those who need it. Despite the importance of this process, the extent to which different patients are offered naloxone has not yet been studied in an ED setting.

### Aims of the study

The present study evaluates a recently-implemented THN program in a busy urban ED to determine the extent to which THN was offered to patients at highest risk of fatal overdose: those who present with a nonfatal opioid overdose. Our specific aims are to measure the proportion of ED visits for opioid overdose in which THN was offered, and identify patient characteristics and other situational variables associated with being offered a THN kit in the ED. Ideally, 100% of individuals who present to the ED with opioid overdose should have an opportunity to leave with a THN kit. However, we predicted that even among this high-risk population, patients with certain demographic or clinical characteristics would have a higher likelihood of being offered THN. Additionally, we anticipated that certain situational variables, such as the time of day, length of stay in the ED, or subsequent hospital admittance, might impact whether clinicians offer THN to patients. By identifying patients that may have been systematically missed, we aimed to develop new insights and recommendations for optimizing the implementation of ED-based THN distribution. Finally, while the focus of our study is whether THN was offered to patients, we also describe the proportion of patients who accepted THN, and any reasons that had been charted for why patients declined THN.

## Methods

### Study design and setting

We conducted a retrospective chart review of all ED visits for which the primary diagnosis was opioid overdose between May 1st, 2016 and April 31st 2017 at the Royal Alexandra Hospital, which is located in Edmonton, Alberta, Canada. This large, urban, tertiary hospital received 73,163 ED visits in 2016–2017 [25]. Additionally, the Royal Alexandra Hospital sees the highest number of ED visits related to opioids and other substances of misuse of any hospital in the province of Alberta, with almost 4000 substance use-related ED visits occurring between 2014 and 2017 [26]. The hospital ED began offering and dispensing THN kits in February 2016. During the study period, patients who were identified by either a physician or a nurse as being at risk for opioid overdose would be offered a THN kit just before they were discharged from the ED. Whether a THN kit was offered was left to the discretion of ED clinicians, as a standardized protocol of which patients to target had yet to be developed at the time of this study.

If the patient accepted the THN kit, a registered nurse would dispense the kit and provide overdose response training, with friends and family included if possible. Registered nurses working in the ED were required to take a training module prior to distributing THN. The kits distributed are publicly funded as part of a province wide THN program, and are provided directly to non-medical persons at no cost and without a prescription. They contain 3 vials of 0.4 mg/ml naloxone, safety-engineered intramuscular syringes, gloves, a CPR face shield, alcohol swabs, and an instructional pamphlet. Kits can be dispensed 24 h per day, 7 days per week. Because THN kits are dispensed directly to the patient from the ED, it is not necessary for patients to fill a naloxone prescription after they leave. Other services for hospital patients who use drugs are available through the Addiction Recovery and Community Health (ARCH) team, a specialty consult service consisting of a multidisciplinary group of physicians with expertise in addiction medicine, nurse practitioners, social workers, addictions counselors, and peer support workers [27]. The ARCH team is available by referral to all ED and hospital inpatients, and offers a combination of harm reduction, in-hospital addiction treatment, health promotion activities, and links to appropriate community health and social supports [27].

### Case identification and data collection

Cases were identified from the patient hospital administrative system according to ICD-10 codes, as any of the following: T40.0-T40.4, and T40.6. We excluded cases if the hospital chart could not be retrieved, or if the patient died in hospital. All medical documents related to patient care were subject to review and data abstraction, including ED physician charts, ED nursing charts, EMS charts, inpatient hospital charts, laboratory reports, and the medication dispensation tracking system. The data abstraction protocol was developed by DD who is an experienced ED nurse, in consultation with KD (emergency medicine specialist) and EH (health services and policy researcher). To establish inter-rater reliability of the abstraction protocol, a second registered nurse independently reviewed a random subset of 70 (20%) medical records. Percent agreement and kappa statistics were calculated for variables collected. For all variables, kappa was above or approaching 0.8, the commonly accepted standard for excellent inter-rater agreement [28].

In addition to reviewing patient charts, each ED visit was linked to data from the provincial Pharmaceutical Information Network (PIN), which tracks prescription medication dispensations. The PIN data were obtained for all opioids dispensed to patients in the 180 days prior to their ED visit for opioid overdose, and included the date each opioid was dispensed, the type of opioid dispensed, and the period it was prescribed for. Our research protocol received ethics approval from the University of Alberta’s Health Research Ethics Board.

### Variables of interest

Our primary outcome of interest was whether THN was offered during their hospital ED visit, or subsequent inpatient hospital stay if admitted. We also determined the number of patients that accepted a THN kit, and reported any reasons that were given by the patient for declining a THN kit. Evidence that THN was offered and accepted was identified in ED physician and nursing charts, as well as inpatient hospital records. Additionally, medication dispensation data were reviewed to confirm cases in which THN was provided.

Patient variables believed to be potentially associated with being offered THN included demographics such as age (per year older) and sex (male vs. female). Additionally, we included several patient characteristics that indicate an increased risk of overdose, such as being a resident of Edmonton’s inner city area (Edmonton Eastwood, yes vs. no), which is the local geographic area with the highest rate of opioid overdose in the city [26]. Other variables linked to overdose risk included having “no fixed address”, which was charted for patients without a permanent address and indicates unstable housing or homelessness [29], (yes vs. no), mental health disorder [13] (defined as bipolar disorder, major depressive disorder, psychosis, personality disorder, or schizophrenia, yes vs. no), and public overdose location [30] (not in a private residence, hotel, healthcare facility or prison, yes vs. no).

Additionally, we examined whether the patient was currently prescribed opioids (yes vs. no), and the type of opioid they had overdosed with, as these characteristics may have impacted clinician perceptions of overdose risk. We identified patients who had a current prescription for any opioid medication at the time of their ED visit by using PIN data to determine whether the prescription period for any opioid dispensed to the patient in the 180 days prior to their ED visit overlapped with their ED visit date.

The patient’s primary opioid intoxicant and route of consumption that caused their opioid overdose was abstracted in the chart review, and was coded into six categories: i) pharmaceutical opioid- oral, ii) pharmaceutical opioid- smoked, iii) pharmaceutical opioid- injected, iv) illegal opioid- oral v) illegal opioid- smoked or vi) illegal opioid- injected. The patient’s primary opioid intoxicant was confirmed by patient self-report, or else suspected by EMS or ED staff, and classified as either illegal or pharmaceutical. Illegal opioids included heroin, carfentanil, and illegally manufactured fentanyl. Fentanyl was assumed to be illegally manufactured if the patient did not have a current prescription for fentanyl at the time of their ED visit, which was determined using PIN data. Pharmaceutical opioids included prescribed fentanyl, and all other pharmaceutical opioids (ie. oxycodone, hydromorphone, morphine) regardless of whether they were prescribed to the patient at the time of their ED visit.

We anticipated that an ED provider’s decision to offer THN may be impacted by the patient’s overdose intention (self-reported by patient, intentional vs. unintentional), as well as the severity of the patient’s overdose. Overdose severity was measured using the patient’s pre-hospital Glasgow Coma Scale (GCS) [3–15], which is a standard test that measures a patient’s eye, motor, and visual responsiveness, with higher scores signifying higher consciousness [31]. GCS was measured by EMS on arrival at the overdose scene, and was therefore only available for patients who arrived at the ED via ambulance.

Several situational variables were examined that could possibly impact whether THN was offered, including admission to hospital (yes vs. no), time of day at presentation and discharge (0:01–8:00, 8:01–16:00, 16:01–24:00), length of ED stay (hours), and whether the visit occurred during a weekend (Saturday or Sunday, yes vs. no). We examined patient disposition, including whether the patient was discharged against medical advice (AMA, yes vs. no), or left ED without treatment or before treatment completion (yes vs. no). A patient was considered to have left the ED AMA if they disclosed to the providers that they intended to leave and signed an AMA form. In contrast, patients who left without treatment or before treatment completion would have registered with triage but then left either from the waiting room or their patient care space, typically without disclosing their intent to leave.

### Analysis

We calculated the number and percentage of each independent variable among ED visits in which THN was offered and not offered. To determine whether each variable was associated with offering THN, we conducted a series of analyses using Generalized Estimating Equations (GEE) regressions for binary outcomes with logit link. Initially, we fitted separate bivariate regression models for each independent variable, with offering THN kits as the outcome. Variables that appeared statistically significant in the initial bivariate analyses at the 0.1-level were included in the final multivariable analysis.

We used GEE with logit link for all regression analyses because a significant portion of patients had multiple ED visits for opioid overdose within our study period, and the data collected for these patients were potentially correlated. With GEE, standard errors are calculated that adjust for multiple observations per patient, in this case using an exchangeable correlation structure [32].

A significant portion of values were missing for pre-hospital GCS (14.9%) and primary opioid intoxicant (23.1%). Missing data for these and other variables were imputed using chained equations with augmented regression and 30 imputations [33]. All variables were included in the imputation regressions, and the outcome was not imputed. We assessed the performance of the multiple imputations by conducting the same multivariate analysis without imputation (Table 3 in Appendix). In this analysis, variables with a high proportion of missingness (GCS, primary opioid intoxicant) contained an additional category for cases with missing values. Analyses were performed using STATA 14.0 IC.

## Results

From May 1st 2016 to April 30th 2017, there were 347 visits to the ED in which the patient received a primary diagnosis of opioid overdose. Among these visits, only 344 patient charts were reviewed, as patient records could not be retrieved for 3 ED visits. Additionally, 2 visits were excluded because the patient died while hospitalized. The remaining 342 ED visits were made by 297 unique patients, of whom 67.3% were males and the mean age was 38.4 (standard deviation: 14.0). Repeat ED visits for opioid overdose were made by 35 patients during the study period, with a range of 1 to 4 ED visits per patient.

**Fig. 1:**
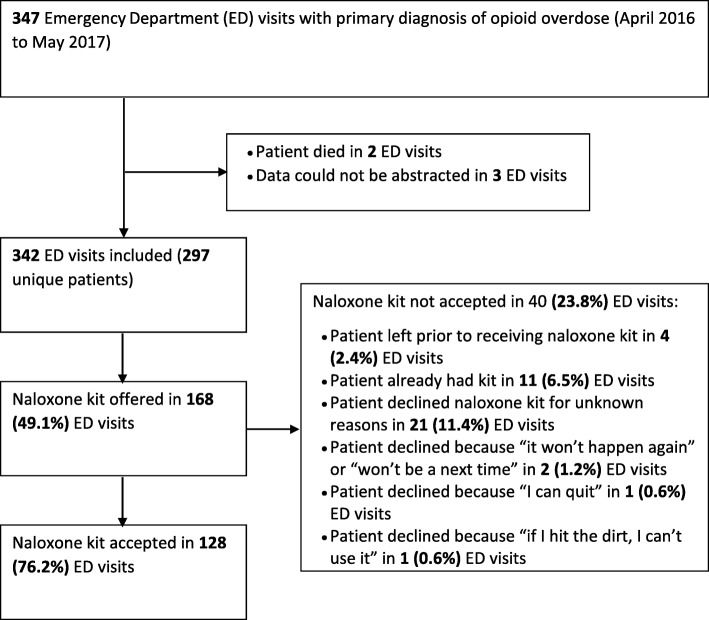
Study flow diagram showing identification of ED visits for inclusion, and the frequencies and percentages of ED visits in which take home naloxone was offered and accepted

Overall, THN was offered to the patient in 168 (49.1%) visits, and was accepted by patients in 128 (76.2%) visits (Fig. 1). Of the ED visits in which the patient accepted THN, a friend or family member was included during the THN training in 31 (24.2%) cases. Among the 40 visits in which the patient declined THN, the patients already possessed a kit in 11 (27.5%) cases, and left the ED prior to receiving naloxone in 4 (10.0%) cases (Fig. 1). Additionally, in two ED visits the patient declined a THN kit because “it won’t happen again,” or “there won’t be a next time.” In one case they stated “I can quit,” and in one instance the patient declined because “if I hit the dirt, I can’t use it” (Fig. 1).

Table 1 shows the breakdown of patient characteristics by ED visit. In 73 (21.4%) ED visits, the patient held a current prescription for an opioid medication at the time of their ED visit. The most common primary opioid intoxicants were smoked illegal opioids (*n* = 66 (25.1%)), injected illegal opioids (*n* = 85 (32.2%)), and pharmaceutical opioids taken orally (*n* = 77 (29.3%)) (Table 1). The patients’ primary opioid intoxicant was confirmed by self-report in 241 (91.6%) visits, suspected by EMS in 8 (3.0%) visits, suspected by ED staff in 12 (4.6%) visits, and confirmed by toxicology screening in 2 (0.8%) visits. Among ED visits in which the patient arrived by ambulance, the patient’s pre-hospital GCS was most frequently in the severe category (GCS 3–8; *n* = 210, 72.2%).

**Table 1:** Characteristics of ED visits for opioid overdose, and associations with offering of take home naloxone kits

Visit Characteristic (*n* = 342)***	Number of ED visits (%)			Unadjusted OR [95% CI]**	*P*-value
	Total (n = 342)	Offered THN (n = 168)	Not offered THN (*n* = 174)		
**Patient characteristics**					
Male sex	234 (68.4)	129 (76.8)	105 (60.3)	2.06 [1.27,3.34]	0.004*
Age (*n* = 341) (Mean, SD)	38.2 (14.0)	35.3 (11.1)	40.9 (15.8)	0.97 [0.96,0.99]	< 0.001*
Resident of inner city^†^ (*n* = 339)	102 (30.1)	50 (30.1)	52 (30.1)	1.04 [0.66,1.65]	0.86
No fixed address (n = 339)^††^	23 (6.8)	12 (7.2)	11 (6.4)	1.03 [0.39,2.67]	0.96
Mental health disorder^¥^	97 (28.4)	37 (22.0)	60 (34.5)	0.62 [0.38,1.01]	0.054*
**Current prescription medications**					
Opioid agonist therapy^§^	9 (2.6)	1 (0.6)	8 (4.6)	0.15 [0.02,0.91]	0.04*
Any current opioid prescription$\mathsf{\varPsi}$	73 (21.4)	21 (12.5)	52 (29.9)	0.37 [0.22,0.65]	< 0.001*
**Overdose details**					
Primary opioid intoxicant (*n* = 263)^a^					
Pharmaceutical opioid- oral^¶^	77 (29.3)	14 (10.5)	63 (48.8)	*ref*	
Pharmaceutical opioid- smoked	7 (2.7)	3 (2.3)	4 (3.1)	3.60 [0.72,18.1]	0.12
Pharmaceutical opioid- injected	16 (6.1)	5 (3.8)	11 (8.5)	2.11 [0.62,7.14]	0.23
Illegal opioid- oral	12 (4.6)	5 (3.8)	7 (5.4)	3.39 [0.91,12.7]	0.070
Illegal opioid- smoked	66 (25.1)	44 (33.1)	22 (16.9)	9.97 [4.66,21.3]	< 0.001*
Illegal opioid- injected	85 (32.3)	62 (46.6)	23 (17.7)	15.1 [6.61,34.3]	< 0.001*
Overdosed in public (*n* = 337)	102 (30.3)	40 (24.1)	62 (36.3)	0.62 [0.39,0.99]	0.043*
Overdose intentional (*n* = 340)	28 (8.2)	6 (3.6)	22 (12.8)	0.30 [0.13,0.65]	0.002*
Glasgow Coma Scale Score (*n* = 291)^a^					
Severe (3–8)	210 (72.2)	127 (88.2)	83 (56.5)	5.60 [2.76,11.3]	< 0.001*
Moderate (9–13)	27 (9.3)	6 (4.2)	21 (14.3)	1.11 [0.37,3.30]	0.85
Mild (14-15)	54 (18.6)	11 (7.6)	43 (29.3)	*ref*	
**EMS & ED care**					
Admitted to hospital	56 (16.4)	15 (8.9)	41 (23.6)	0.35 [0.19,0.65]	0.001*
Left against medical advice^‡^	34 (9.9)	14 (8.3)	20 (11.5)	0.72 [0.33,1.57]	0.40
Left without treatment/before treatment complete^‡‡^	29 (8.5)	6 (3.6)	23 (13.2)	0.19 [0.07,0.53]	0.002*
Time day at ED presentation^b^					
0:01–8:00	94 (27.5)	47 (28.0)	47 (27.0)	*ref*	
8:01–16:00	108 (31.6)	49 (29.2)	59 (33.9)	0.92 [0.54,1.59]	0.77
16:01–24:00	140 (40.9)	72 (42.9)	68 (39.1)	1.10 [0.65,1.84]	0.73
Time of day at discharge^c^					
0:01–8:00	121 (35.4)	58 (34.5)	63 (36.2)	*ref*	
8:01–16:00	94 (27.5)	53 (31.5)	41 (23.6)	1.43 [0.82,2.50]	0.21
16:01–24:00	127 (37.1)	57 (33.9)	70 (40.2)	0.94 [0.55,1.59]	0.82
ED visit during weekend	102 (29.8)	49 (29.2)	53 (30.5)	0.99 [0.61,1.59]	0.96
Length of ED stay (median, IQR)	5.5 (3.3,9.2)	4.52 (2.7,7.6)	6.45 (3.9,11.0)	0.98 [0.95,1.01]	0.20

*** N = 342 for all independent variables unless stated otherwise

** Odds ratio of being offered take home naloxone for each independent variable

* Statistically significant at the level of 0.1 and thus eligible for inclusion in multivariable analysis

a- Overall *P*-value < 0.001

b- Overall *P*-value =0.77

c- Overall *P*-value = 0.26

† Postal code overlaps Local Geographical Area of Edmonton Eastwood, includes T5B, T5G, T5H, T5J, T5K, T6W

†† Postal code was charted as “no fixed address,” indicating that the patient is likely unstably or homeless

¥ Anxiety disorder, Bipolar disorder, Major Depressive Disorder, Psychosis, Personality Disorder (Axis II), or Schizophrenia)

§ Methadone (liquid form, once daily ingestion, max period of 7 days), or Suboxone (once daily dosing, max period of 7 days)

$\mathsf{\varPsi}$ Does not include prescriptions for opioid agonist therapy

¶ Oral includes oral (97%), rectal (1%), transdermal (1%), and Percutaneous endoscopic gastrostomy (1%)

‡ disclosed to the providers that they intended to leave and signed an AMA form

‡‡ registered with triage and was assessed, but then left without warning or disclosing their intent to leave

Table 1 shows the results of the initial unadjusted bivariate analyses. We found that patients were less likely to be offered THN if they overdosed in a public location (OR = 0.62 [0.39,0.99], intentionally overdosed (OR = 0.30 [0.13,0.65]), left the ED without treatment or before treatment completion (OR = 0.19 [0.07,0.53]), or were admitted to hospital (OR = 0.35 [0.19,0.65]), (Table 1). Additionally, older patients were less likely to be offered THN in bivariate analysis (OR = 0.97 [0.96,0.99], per year older), (Table 1). In contrast, patients were more likely to be offered THN if they had a severe GCS score upon EMS arrival (OR = 5.60 [2.76,11.3]) for GCS 3–8 vs. 14–15), had smoked an illegal opioid (OR = 9.97 [4.66,21.3] for illegal opioid-smoked vs. pharmaceutical opioid- oral), or had injected an illegal opioid (OR = 15.1 [6.61,34.3] for illegal opioid- injected vs. pharmaceutical opioid- oral), (Table 1).

Table 2 shows the results of the multivariable analysis. Variables that were independently and positively associated with being offered THN included having a severe overdose as measured by GCS (AOR = 3.70 [1.63,8.37] for GCS 3–8 vs. GCS 14–15), and smoking or injecting an illegal opioid (AOR = 3.78 [1.32,10.9] and AOR = 6.05 [2.15,17.0] respectively for illegal opioid- smoked vs. pharmaceutical opioid- oral and illegal opioid-injected vs. pharmaceutical opioid- oral), (Table 2). In contrast, patients were less likely to receive a THN kit if they left the ED without treatment or before treatment completion (AOR = 0.16 [0.05,0.48]), if they were admitted to hospital (AOR = 0.46 [0.22,0.97]) or if they had an opioid prescription at the time of their ED visit (AOR = 0.41 [0.19,0.88]).

**Table 2:** Multivariable associations of patient characteristics with offering of take home naloxone during ED visits for opioid overdose, with multiple imputation

Visit Characteristic (*n* = 342)	Adjusted OR [95% CI]**	*P*-value
Male sex	1.75 [0.97,3.16]	0.064
Age	1.00 [0.98,1.02]	0.92
Mental Health disorder	1.12 [0.59,2.12]	0.74
Opioid agonist therapy	0.37 [0.04,3.35]	0.37
Any current opioid prescription at time of ED visit	0.41 [0.19,0.88]	0.021*
Primary opioid intoxicant illegal†		
Pharmaceutical opioid- oral	*ref*	
Pharmaceutical opioid- smoked	1.32 [0.24,7.27]	0.75
Pharmaceutical opioid- injected	1.40 [0.36,5.51]	0.63
Illegal opioid- oral	1.39 [0.33,5.88]	0.66
Illegal opioid- smoked	3.78 [1.32,10.9]	0.014*
Illegal opioid- injected	6.05 [2.15,17.0]	0.001*
Overdosed in public	0.61 [0.33,1.11]	0.11
Overdosed intentionally	0.59 [0.21,1.62]	0.30
Pre-hospital GCS††		
Severe (3–8)	3.70 [1.63,8.37]	0.002*
Moderate (9–13)	2.09 [0.61,7.21]	0.24
Mild (14, 15)	*ref*	
Left without treatment/ before treatment completion	0.16 [0.05,0.48]	0.001*
Admitted to hospital	0.46 [0.22,0.97]	0.040*

†Overall p-value = 0.009

††Overall p-value = 0.008

*Statistically significant at the level of 0.05

**Odds ratio of being offered take home naloxone for each independent variable, adjusted for all other variables in the model

The results of the multivariate analysis conducted without multiple imputation are similar to those generated with imputations (Table 3 in Appendix ). In particular, the same variables were statistically significant in both models, with the one exception being that male sex was found to be significantly associated with being offered THN in the non-imputed multivariate model (Table 3 in Appendix).

## Discussion

In this evaluation of a recently implemented ED-based THN program, THN was offered to patients in approximately half of ED visits for opioid overdose. We sought to determine whether a recently implemented ED-based THN program was missing certain patients by identifying patient characteristics and other situational variables associated with being offered THN. We found that patients were more likely to be offered THN if they experienced a severe overdose (GCS of 3–8), or had consumed an illegal opioid. In contrast, patients who had an active opioid prescription at the time of their ED visit, who left the ED without treatment of before treatment completion, or who were admitted to the hospital were less likely to be offered THN.

The proportion of patients offered THN (49%) is significantly higher than in previous studies which have evaluated the implementation of ED-based THN programs [23, 34]. This higher rate is likely attributable to our decision to only include individuals who were discharged with a diagnosis of opioid overdose. This decision was made based on our desire to examine whether the THN program was reaching the highest risk patients. These patients are likely to be readily identifiable by ED providers, and thus more likely to be offered THN compared to other patient groups at risk of overdose. In previous qualitative studies about ED-based THN programs, ED providers frequently acknowledged that it is important not to miss patients who have visited the ED for an opioid overdose [23, 24].

Our results showed that even among patients who have recently overdosed, certain patients were more likely to be offered than others. It is likely that in the context of a busy ED, a clinician’s decision to offer THN may be driven by their perceptions of which patients are most at risk for overdose death. For instance, ED staff may more readily offer THN to patients who have experienced a severe overdose because they are easily recognized as being at risk for future overdose death. Similarly, clinicians may more readily recognize people who smoke or inject illegal drugs as being more vulnerable for future overdose. This tendency is likely shaped by both clinical experience and media reports, as it is true that in the Western Canadian provinces of Alberta and British Columbia, over 80% of accidental opioid overdose deaths involved illegally manufactured fentanyl in 2017 [26, 35]. Additionally, clinicians may believe that THN is predominately meant for people who use illegal drugs, given that THN was originally developed to serve people who inject heroin [36]. Indeed, the initial rationale for THN programs was in-part to empower people who use heroin who were reluctant to call an ambulance in cases of overdose for fear of criminal prosecution [37].

Our finding that patients who were taking prescription opioids were less likely to be offered THN is consistent with previous reports. In a survey of Canadian ED physicians, it was found that while the large majority of participants (> 90%) agreed or strongly agreed that patients with a history of emergency care for opioid overdose would benefit from THN programs, fewer physicians (69%) agreed that patients prescribed high doses of opioids would benefit [19]. Similarly, previous qualitative studies found that almost all ED staff who were interviewed agreed certain patients should receive THN—such as those who are have overdosed in the past, are opioid dependent, or who inject opioids [23, 24]. However, other staff disagreed on whether it was appropriate, necessary, or realistic to offer THN to all patients prescribed opioids [23, 24]. In some cases, this appeared be due to their perceived lower risk of overdose [23, 24].

Specific reasons as to why clinicians may be less likely to provide THN to patients taking prescription opioids have been explored in studies of primary care patients with chronic pain [38, 39]. For instance, in one qualitative study, primary care providers believed that co-prescribing THN may offend patients due to the stigma associated with substance use disorders and THN [38]. Previous research from primary care has generally refuted this perception. Indeed, the majority of patients on long-term opioid prescriptions give either positive or neutral reactions to being offered a naloxone prescription, report wanting naloxone prescriptions in the future, and agree that THN should be available to patients prescribed opioids for pain [40]. Similarly, only about 13% of patients with chronic non-cancer pain report that they would be offended if offered THN [41].

Other primary care providers have expressed reluctance to co-prescribe THN with opioids because they felt that THN should not be necessary if opioids are prescribed properly [38]. However, offering THN to patients prescribed opioids can facilitate important conversations about overdose risk, and may even change behavior in ways that reduce the risk of future overdose and ED visits [40, 42]. For instance, at least some patients who received THN and overdose education through primary care report safer dosing, safer timing, and increased knowledge of opioids and overdose [40]. Additionally, a non-randomized trial of THN co-prescriptions in primary care found that patients who received THN had 63% less ED-related visits at 12-months compared to those that did not receive THN [42].

It was not surprising that patients who unexpectedly left the ED without treatment or before treatment completion were less likely to be offered THN. At the time of this study, ED staff typically waited until discharge to offer THN to patients. Therefore, patients who left the ED without disclosing their intent to leave may have been missed. Patients who have been treated for opioid overdose may be experiencing symptoms of withdrawal and be eager to leave the ED to address these symptoms. Consequently, they may be less willing to complete discharge paperwork or THN training [23]. These patients may be especially vulnerable to a subsequent overdose immediately following their ED visit, given the relatively short half-life of naloxone [43], and the additional risk posed by consuming further doses of opioids after leaving the ED. Equipping these patients with THN (and offering opioid agonist treatment and other supports) is especially critical. Finally, our findings showed that ED patients who were admitted to the hospital were more likely to be missed. Further efforts are needed to expand THN distribution to at-risk hospital inpatients in this setting.

The majority (82%) of patients in our study who were offered a THN kit either accepted it or already possessed one, which confirms previous reports showing that ED-based naloxone distribution is acceptable to patients. This THN acceptance rate was slightly higher than the 68% reported in a previous study by *Kestler* et al.*,* in which THN was offered to patients who had reported illegal drug use, were prescribed a high dose of prescription opioids, were receiving opioid agonist therapy, or had any clinical presentation suggestive of opioid use [20]. The higher acceptance rate among our population may suggest that people who have recently experienced an opioid overdose are more accepting of THN than other THN-eligible patients. In a minority of cases, the patient’s reason for declining a THN kit had been recorded. The reasons included feeling that they were no longer at risk, intending to stop using drugs, and believing that THN is not useful if opioids are used alone. Several of these reasons are consistent with a previous evaluation of ED patient acceptance of THN, which found that the patient beliefs of being “not at risk” and “done with drugs” were the two most common rationales given for declining THN [44].

### Limitations

There are several limitations to this study that are inherent to retrospective chart reviews. Because medical records are created for clinical purposes, some information may not be charted consistently. In particular, we were unable to ensure that every instance of THN being offered was documented. We attempted to ameliorate this issue by reviewing medication dispensation data to confirm cases in which THN was both offered and accepted by the patient. However, for some cases in which THN was offered but declined by the patient, ED providers may not have charted that they offered THN. Therefore, the proportion of ED visits in which THN was offered may have been underestimated.

Other variables were missing values for a significant percentage of ED visits, including pre-hospital GCS (14.9%), and primary opioid intoxicant (23.1%). We attempted to account for the uncertainty created by missing data with multiple imputation. We obtained similar results from the multivariate analysis with and without the use of multiple imputation (Table 3 in Appendix).

The data may not always be accurate for variables that were partially or fully based on patient self-report or clinician suspicion, such as the patient’s primary intoxicant. Patients may have been reticent to disclose the complete details related to their substance use, or they may have consumed a different substance from what they believed. However, given that the aim of the study was to examine clinician behavior, measuring the clinicians’ perception of the patients’ opioid intoxicant is likely more useful than the actual intoxicant.

Other variables that were not collected may have also impacted a clinician’s decision to offer THN. For instance, we were unable to obtain reliable data on whether patients had been prescribed or had consumed any benzodiazepines. It is possible that ED providers were more likely to offer THN to patients who used opioids in combination with benzodiazepines, given that this combination is a strong risk factor for overdose among people receiving opioid analgesics [45, 46].

The data abstracters were not blinded to the purpose of the study. However, it is unlikely this affected the results significantly because there were no specific a priori hypotheses regarding which variables would be associated with THN being offered. Additionally, because our sample was limited to a single hospital site, it is possible that our results may not generalize to other geographic locations.

While we have reported on reasons that patients gave for declining THN, these reasons were only recorded for a minority of patients. Consequently, it was not possible to draw conclusions based on this data. Finally, this study only captured a small subpopulation of individuals visiting the ED who are potentially at risk for overdose. Future research is needed to evaluate whether ED providers are able to reach other patient groups at risk for overdose.

## Conclusions

This study is the first to identify patient characteristics associated with being offered THN among a high risk population of ED patients. Our findings add to the current literature by demonstrating that implementation of an ED-based THN program and accompanying staff training does not necessarily guarantee that even patients presenting with opioid overdose will be offered THN. In the absence of a standardized protocol to identify high risk patients, ED providers may consciously or unconsciously rely on their own perceptions as to who is at risk and overlook certain patients, such as those who use prescription opioids. To ensure optimal implementation of ED-based THN programs, ED staff should be provided with information on the importance of offering THN widely, and clear guidelines regarding which patients should receive THN. Further, administrators or clinicians implementing ED-based THN programs should establish a reliable inpatient pathway to provide THN for eligible patients prior to discharge. As we have shown, patients admitted to the hospital after receiving acute care for an opioid overdose can be missed by ED-based THN programs.

Another way for ED-based THN programs to improve their coverage to at-risk patients is to avoid waiting until discharge to offer THN. As discussed, our evaluation of an ED-based THN program showed that patients who left the ED unexpectedly were more likely to be missed by providers. Where possible, THN should be offered earlier in the patient’s ED visit, to ensure they are not missed should they leave the ED unexpectedly.

At minimum, all patients presenting to an ED with an opioid overdose should be offered THN. However, several other groups would likely also benefit from THN distribution and training in an ED setting. In particular, the Center for Disease Control and Prevention recommends that THN be offered to patients taking higher doses of prescription opioids (≥50 OME/day), patients with concurrent opioid and benzodiazepine use, and patients with a history of a substance use disorder [47]. We suggest that in addition to targeting people who use opioids, ED providers should consider offering THN to people who use any illegal substance, including stimulants. People who use non-opioid illegal substances may be exposed to opioids through contamination [48], or may witness an opioid overdose among their peers. Future evaluations are needed to examine the extent to which these different patients can be targeted, and to develop systematic protocols to identify various individuals at risk of overdose. One such intervention that holds promise is electronic health record prompts, which can be triggered by terms entered into the patient’s initial assessment [49, 50]. Such interventions have been previously demonstrated to increase the distribution of THN to ED patients discharged after opioid overdoses [49, 50]. As evidenced by our study, without such systematic approaches, attaining complete coverage of even the highest risk patients is difficult when implementing ED-based THN programs.

## Data Availability

The datasets analyzed during the current study are available from the corresponding author on reasonable request.

## Appendix

**Table 3:** Multivariable associations of patient characteristics with offering of take home naloxone during ED visits for opioid overdose, without multiple imputation

Visit Characteristic (*n* = 334)	Adjusted OR [95% CI]**	*P*-value
Male sex	1.94 [1.10,3.41]	0.022*
Age (n = 341)	1.00 [0.98,1.02]	0.93
Mental Health disorder	1.15 [0.61,2.15]	0.67
Opioid agonist therapy	0.30 [0.04,2.44]	0.26
Any current opioid prescription at time of ED visit	0.44 [0.21,0.92]	0.029*
Primary opioid intoxicant (*n* = 263)†		
Pharmaceutical opioid- oral	*ref*	
Pharmaceutical opioid- smoked	1.14 [0.25,5.26]	0.87
Pharmaceutical opioid-injected	1.43 [0.34,6.01]	0.63
Illegal opioid- oral	1.26 [0.33,4.83]	0.74
Illegal opioid- smoked	3.65 [1.35,9.88]	0.011*
Illegal opioid- injected	5.47 [2.07,14.5]	0.001*
Missing	1.61 [0.63,4.08]	0.32
Overdosed in public (*n* = 337)	0.64 [0.35,1.15]	0.13
Overdosed intentionally (*n* = 340)	0.47 [0.17,1.31]	0.15
Pre-hospital GCS (*n* = 291)††		
Severe (3–8)	3.57 [1.62,7.83]	0.002*
Moderate (9–13)	1.39 [0.39,4.98]	0.62
Mild (14, 15)	*Ref*	
Missing	3.64 [1.40,9.42]	0.008*
Left without treatment/ before treatment completion	0.18 [0.06,0.57]	0.003*
Admitted to hospital	0.40 [0.19,0.83]	0.014*

†Overall P-value 0.003

††Overall p-value 0.006

*Statistically significant at the level of 0.05

**Odds ratio of being offered take home naloxone for each independent variable, adjusted for all other variables in the model

## Abbreviations

AMA: Against Medical Advice
AOR: Adjusted Odds Ratio
ARCH: Addiction Recovery and Community Health
ED: Emergency Department
EMS: Emergency Medical Service
GCS: Glasgow Coma Scale
GEE: Generalized Estimating Equation
OR: Odds Ratio
PIN: Pharmaceutical Information Network
THN: Take Home Naloxone
